# Clinical Significance of Pre-Transplant Arterial Stiffness and the Impact of Kidney Transplantation on Arterial Stiffness

**DOI:** 10.1371/journal.pone.0139138

**Published:** 2015-09-25

**Authors:** Hyun Seon Kim, Jaeho Seung, Ju Hyun Lee, Byung Ha Chung, Chul Woo Yang

**Affiliations:** 1 Transplant Research Center, Seoul St. Mary's Hospital, College of Medicine, The Catholic University of Korea, Seoul, Korea; 2 Division of Nephrology, Department of Internal Medicine, Seoul St. Mary's Hospital, College of Medicine, The Catholic University of Korea, Seoul, Korea; Mario Negri Institute for Pharmacological Research and Azienda Ospedaliera Ospedali Riuniti di Bergamo, ITALY

## Abstract

**Background:**

Arterial stiffness is closely associated with cardiovascular disease (CVD) in end stage renal disease (ESRD) patients. However, the clinical significance of pre-transplant arterial stiffness and the impact of kidney transplantation (KT) on arterial stiffness have not yet been determined.

**Method:**

We measured the brachial-ankle pulse wave velocity (baPWV) before KT and one year after KT. We evaluated the potential utility of pre-transplant baPWV as a screening test to predict CVD. The impact of KT on progression of arterial stiffness was evaluated according to changes in baPWV after KT. The factors that influence the change of baPWV after KT were also examined.

**Result:**

The mean value of pre-transplant baPWV was 1508 ± 300 cm/s in ESRD patients; 93.4% had a higher baPWV value than healthy controls. Pre-transplant baPWV was higher in patients with CVD than in those without CVD (1800 ± 440 vs. 1491 ± 265 cm/s, p<0.05), and was a strong predictive factor of CVD (OR 1.003, p<0.05). The optimal cut-off value of baPWV for the detection of CVD was 1591 cm/s, and this value was an independent predictor of CVD in KT recipients (OR 6.3, p<0.05). The post-transplant baPWV was significantly decreased compared to that of pre-transplant rates (1418 ± 235 vs. 1517 ± 293 cm/s, p<0.05), and progression of arterial stiffness was not observed in 86.9% patients. Logistic regression analysis revealed that higher body mass index and the degree of increase in calcium levels were independent risk factors that affected baPWV after KT.

**Conclusions:**

Evaluation of arterial stiffness with baPWV is a useful screening test for predicting CVD after KT, and KT is effective in preventing the progression of arterial stiffness in ESRD patients.

## Introduction

Cardiovascular disease (CVD) is the leading cause of death in end stage renal disease (ESRD) patients [1, 2]. Compared to the general population, ESRD patients have a higher frequency of the more traditional risk factors, such as dyslipidemia, diabetes, and old age, as well as additional vascular risk factors, such as endothelial dysfunction, vascular inflammation, and calcification. Kidney transplantation (KT) is effective in reducing CVD risk factors in ESRD patients, although CVD is still one of the main causes of death in KT recipients [3, 4].

Increased arterial stiffness is very common and is associated with an increased risk of CVD in ESRD patients [5]. The evaluation of arterial stiffness by measuring brachial-ankle pulse wave velocity (baPWV) in ESRD patients is one of the recommended approaches in predicting CVD risk after KT [4, 6, 7]. However, the effects of KT on arterial stiffness have shown discrepant results likely related to the different study populations examined, evaluation time points, and follow-up periods after KT [8–10].

Therefore, the present study was designed to assess the utility of arterial stiffness measurements as an early marker in predicting CVD in KT recipients. The results of our study clearly demonstrate that baPWV measurement is a useful tool for predicting CVD after KT in ESRD patients.

## Materials and Methods

### Study Population

A total of 171 ESRD patients for eligible for KT at the Seoul Mary’s Hospital from January 2011 to December 2013 were enrolled in this study. The clinical characteristics and biochemical parameters are described in Table 1. Forty-one patients (24.0%) underwent preemptive KT. The majority of patients (94.2%) were treated with tacrolimus and others were treated with cyclosporine (5.8%) in conjunction with mycophenolic acid and steroids. All patients were followed for 33.9 ± 6.1 months, and CVD occurred in 10 patients (ST segment elevation myocardial infarction (STEMI) [n = 1], non-ST segment elevation myocardial infarction [n = 3], angina with significant electrocardiography (ECG) change [n = 3], peripheral artery disease (PAD) [n = 3]). Follow-up baPWV after KT was available in 84 patients. Healthy controls were taken from a previous study in which the baPWV measuring machine (VP-1000 BP203RPEII) was used to set up reference values. A total of 12,517 healthy subjects (4,488 men and 3,393 women, aged 25–87 years) were recruited from the Tokyo Medical University Hospital for this study, and their baPWV measures were considered as the standard values of the general population [11].

**Table 1:** Baseline demographics and hemodynamic parameters in end stage renal disease patients.

	All (n = 171)	CVD Event (n = 10)	No CVD event (n = 161)	p-value
Male (%)	53.8	50.0	54.0	0.772
CVD history (%)	5.8	30	4.3	<0.05
Age (year)	44.5 ± 11.6	51.2 ± 6.4	43.6 ± 11.6	<0.05
BMI (%)	23.1 ± 3.5	25.9 ± 3.1	23.0 ± 3.5	<0.05
Dialysis duration (month)	32.6 ± 6.1	18.1 ± 27.4	33.4 ± 107.4	0.612
Hb (g/dL)	9.9 ± 1.8	10.0 ± 1.3	9.9 ± 1.9	0.861
Ca (mg/dL)	8.5 ± 1.0	8.8 ± 1.1	8.5 ± 1.0	0.394
Ph (mg/dL)	5.1 ± 1.5	5.6 ± 1.7	5.1 ± 1.4	0.286
iPTH (pg/mL)	229 ± 197	131 ±106	238 ± 200	0.286
CRP (mg/L)	0.8 ± 1.9	0.22 ± 0.2	0.85 ± 2.0	0.374
TC (mg/dL)	154 ± 37	163 ± 49	153 ± 36	0.414
TG (mg/dL)	127 ± 71	188 ± 138	124 ± 75	<0.05
LDL (mg/dL)	81.2 ± 26.7	85.3 ± 27.6	80.7 ± 26.7	0.606
HDL (mg/dL)	39.7 ± 15.4	37.3 ± 10.9	39.8 ± 15.8	0.624
25(OH)D (ng/mL)	10.1 ± 6.0	8.4 ± 5.1	10.1 ± 5.9	0.936
Pulse pressure (mmHg)	54.2 ± 13.4	71.3 ± 10.1	53.1 ± 12.7	<0.05
SBP (mmHg)	139.0 ± 21.6	162.8 ± 21.3	137.5 ± 20.6	<0.05
DBP (mmHg)	84.3 ± 12.8	94.5 ± 14.2	83.9 ± 12.7	0.072
EF (%)	61.0 ± 5.2	58.8 ± 4.0	61.2 ± 5.2	0.154
LVMI (g/m^2^)	145.0 ± 38.2	173.5 ± 54.7	143.4 ± 35.9	<0.05

CVD, cardiovascular disease; BMI, body mass index; Hb, hemoglobin; Ca, calcium; Ph, phosphate; iPTH, intact parathyroid hormone; CRP, C-reactive protein; TC, Total cholesterol; TG, triglyceride; LDL, low density lipoprotein; HDL, high density lipoprotein; 25(OH)D, 25-hydroxyvitamin D; SBP, systolic blood pressure; DBP, diastolic blood pressure; EF, ejection fraction; LVMI, left ventricle mass index

### Study Design

This study was approved by the Institutional Review Board (IRB) of the Seoul St. Mary’s Hospital (KC15RISI0363). This study is retrospective, and the medical records were collected during the medical treatment, not for research, so we were exempted from acquirement of consent by IRB. Patient records were anonymized and unidentified prior to analysis.

Patients underwent baPWV evaluation within 2 months prior to KT and approximately 1 year after KT. First, we evaluated the role of pre-transplant baPWV in predicting CVD after KT, and receiver operating characteristic (ROC) curve analysis was used to determine the best baPWV cut-off value able to predict CVD. Second, we evaluated the effects of KT on baPWV changes and factors influencing changes in baPWV. Third, we evaluated the impact of KT on arterial stiffness in the clinical setting. Patients were divided into four groups according to the severity of arterial stiffness, and the progression of arterial stiffness after KT was evaluated in each group.

We reviewed patients’ medical records and collected their baseline characteristics including age, sex, past history, dialysis duration, blood pressure (BP), left ventricular mass index (LVMI), ejection fraction (EF) in echocardiography, and laboratory findings. In addition, we evaluated factors that could affect arterial stiffness, including a comparison of the coronary calcification score and pulse pressure with baPWV.

### Measurement of Pulse Wave Velocity

Patients rested in a supine position for 5 minutes, and baPWV was measured at the brachial and tibial arteries. The baPWV was recorded with a VP-1000 BP203RPEII (Colin Company, Kyoto, Japan), which simultaneously recorded both systolic blood pressure (SBP) and diastolic blood pressure (DBP), and registered the electrocardiogram over the course of 15 minutes. Patients were instructed not to take any medications, caffeine, or alcohol on the day of the examination. Waveforms were evaluated from plethysmographic sensors placed in cuffs on both upper arms and on both ankles to reflect the brachial artery and tibial artery values. The instrument automatically recorded the time intervals between the wave at the right upper arm and both ankles, and the distance between the upper arms and both ankles. The baPWV (cm/sec) calculated the distance between the two recording points divided by the time interval. This device automatically and simultaneously measures bilateral baPWV and brachial and ankle blood pressure. The pulse pressure (mmHg) was calculated as the difference between SBP and DBP.

In our analysis, we used the mean value of the bilateral baPWV. On the basis of baPWV reference values from healthy controls of the same age and sex, patients were grouped into ‘normal’, ‘hardish’, ‘slightly harder’, ‘and harder’ groups. By comparing the values of baPWV before and after KT within these groups, we evaluated the progression of arterial stiffness.

### Measurement of Coronary Calcium Scores

Calcification of a coronary artery indicates the presence of atherosclerotic plaque in the blood vessel, and the coronary calcium score is a well-known predictor of CVD in ESRD patients [2, 12, 13], and has been used as a screening test for the evaluation of cardiovascular status before KT. In this study, we tested whether baPWV is comparable to the coronary Calcium score as a screening test. Briefly, the coronary Calcium score can be calculated by measuring the amount of Calcium in the walls of the arteries that supply the heart muscle using a high resolution computed tomography (CT) scan of the chest. We measured the coronary Calcium score at the time of measuring baPWV before KT. The Coronary Calcium score was categorized as low and high based on the standard value of 100, according to the risk of both major coronary events and plaque burden [12].

### Measurement of Transthoracic Echocardiography

Pre-transplant echocardiography was performed at rest, and using commercially available ultrasound system (Vivid 7, GE; Vingmed Ultrasound, Horton, Norway; Sequoia 512, Acuson, Mountain View, CA). All of the measurements were collected using the standard methods specified in the guidelines of the American Society of Echocardiography (ASE). All of the M-mode measurements for calculating left ventricular mass (LVM) were made during end-diastolic, and the LVM was divided by body surface area to calculate the left ventricular mass index (LVMI).

### Biochemical Parameters

Data for hemoglobin (Hb), serum calcium (Ca), serum phosphate (Ph), serum intact PTH (iPTH), total cholesterol (TC), high density cholesterol (HDL), low density cholesterol (LDL), triglycerides (TG), 25-hydroxyvitamin D (25(OH)D), and C-reactive protein (CRP) were obtained at the time of measuring baPWV before KT and after KT using standard methodology. We calculated the difference of these values before and after KT, and called it the delta value.

### Statistical Analysis

Statistical analysis was performed with Statistical Package for the Social Sciences (SPSS) software, version 15.0 (SPSS Inc., Chicago, Ill., USA). Data were expressed as mean ± standard deviation and p<0.05 was considered statistically significant. Differences between groups were assessed by the Wilcoxon rank sum, *t*-test. Correlations were assessed by the Spearman correlation index; Linear and binary logistic regression analyses were used to determine the parameters associated with baPWV, and the regression coefficient and an odds ratio was measured when a logistic regression was calculated. To determine the cut-off values for baPWV as a predictor of CVD, ROC curve analysis was used.

## Results

### Role of Pre-transplant baPWV in Predicting Cardiovascular Disease after Kidney Transplantation

**Fig 1:**
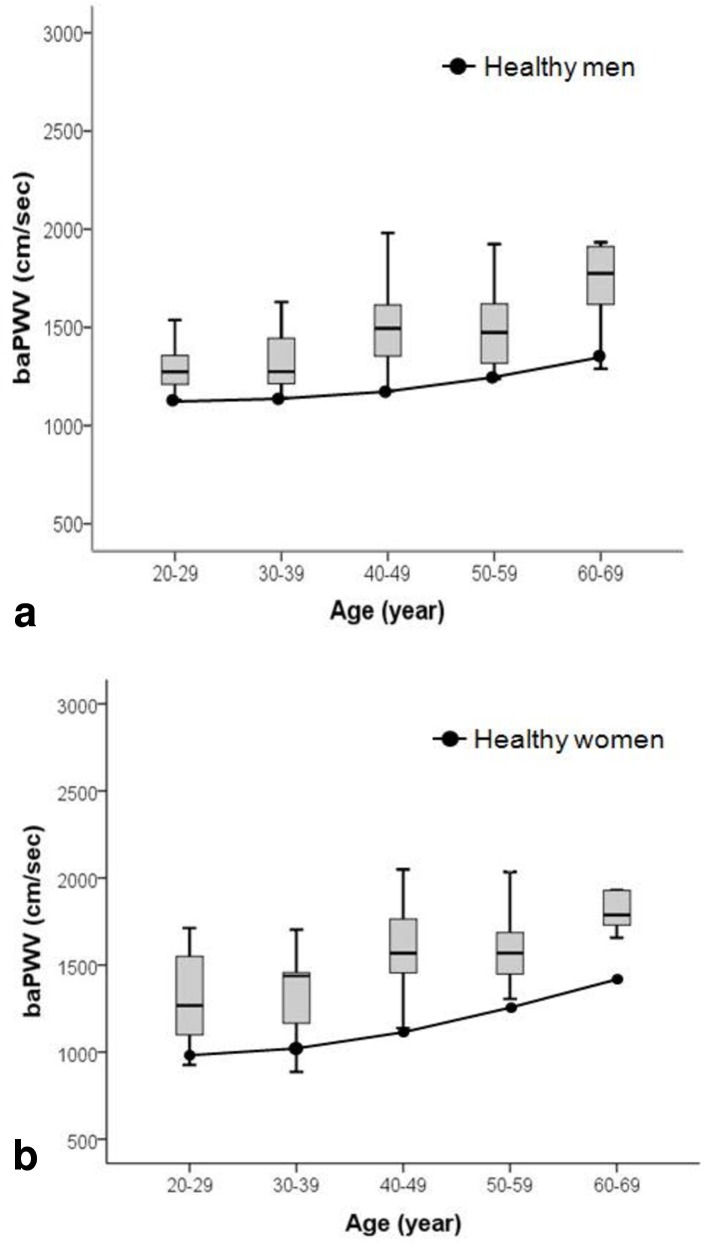
The reference value of brachial-ankle pulse wave velocity in healthy controls and end stage renal disease patients. (A) The mean value of pre-transplant baPWV in end stage renal disease patients in men. The mean value of healthy men presented as linear chart (B) The mean value of pre-transplant baPWV in end stage renal disease patients in women. The mean value of healthy women presented as linear chart. Note that the mean value of pre-transplant baPWV in end stage renal disease patients was much higher than healthy controls.

**Fig 2:**
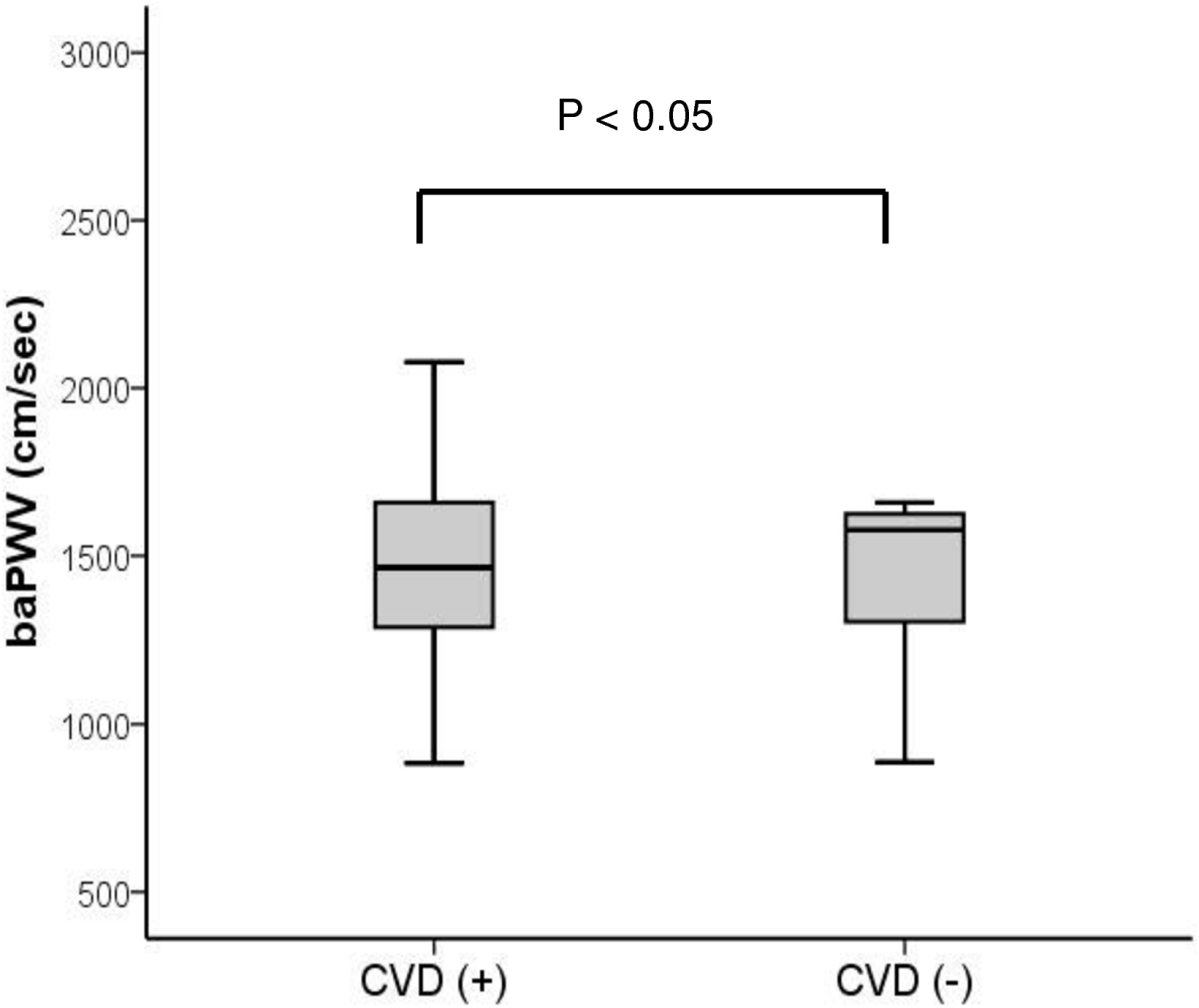
The comparison of pre-transplant brachial-ankle pulse wave velocity in patients with and without cardiovascular disease after kidney transplantation. Note that pre-transplant baPWV in patients with CVD was higher than that of patients without CVD. baPWV = brachial-ankle pulse wave velocity; CVD = cardiovascular disease.

The mean value of the baPWV was 1508 ± 300 cm/s in ESRD recipients, and 93.4% had a higher baPWV than healthy controls with same age and sex in the Asian population (Fig 1A and 1B). CVD developed in ten patients in ESRD recipients, and the pre-transplant baPWV was higher in patients with CVD than those of other patients without CVD (1800 ± 440 vs. 1491 ± 265 cm/s, p<0.05, Fig 2), and it also proved to be a strong predictive factor of CVD (OR 1.003, CI: 1.001–1.005, p<0.05) in binary logistic regression analysis.

**Fig 3:**
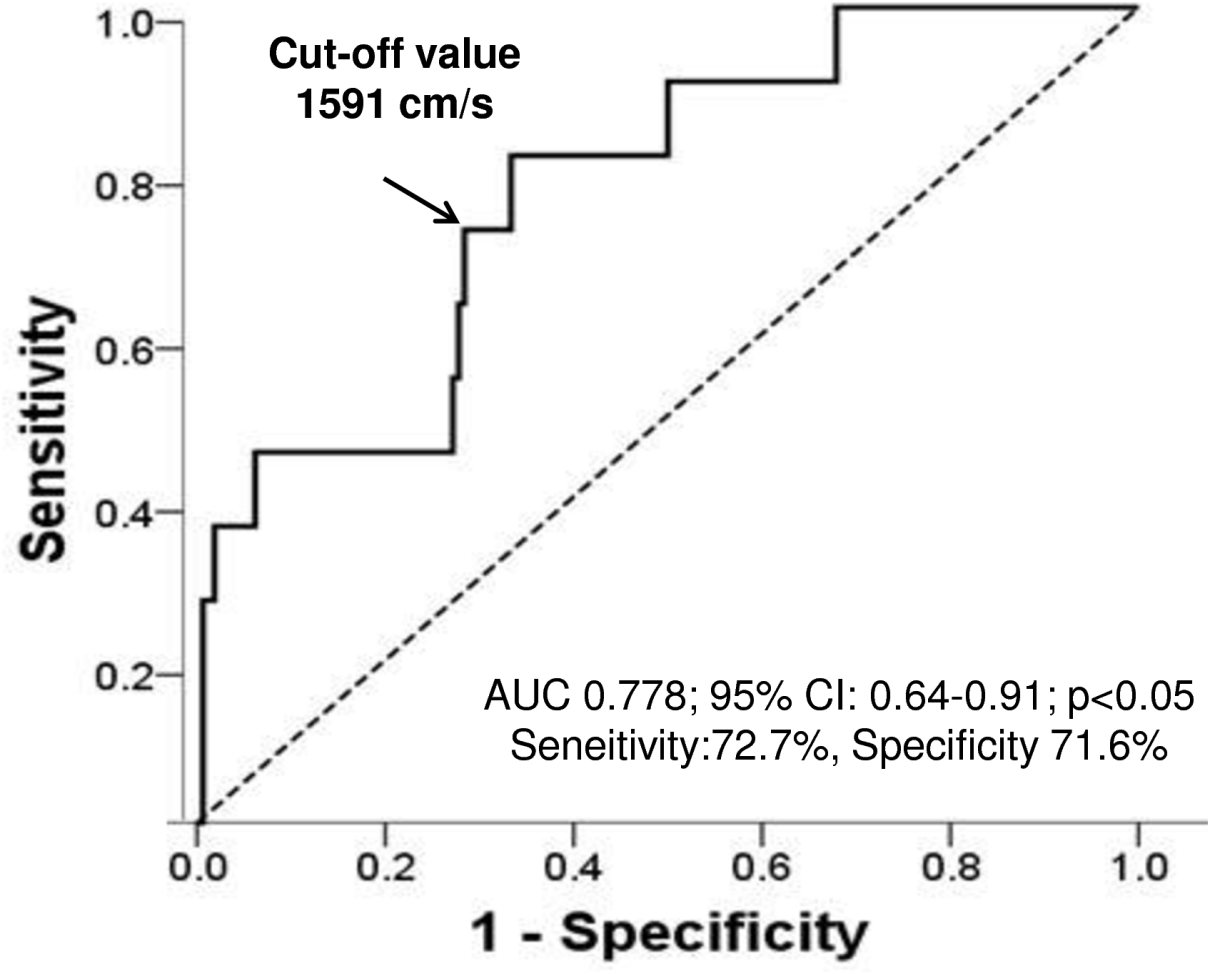
Receiver-operating characteristic curve analysis to assess best cut-off value for prediction of cardiovascular disease by brachial-ankle pulse wave velocity. ROC curves for detecting CVD by using baPWV in end stage renal disease patients. The AUC is a measure of how well baPWV can distinguish between patients with and without CVD. ROC = receiver-operating characteristic; CVD = cardiovascular disease; baPWV = brachial-ankle pulse wave velocity; AUC = area under the curve.

In the ROC curve analysis (Fig 3), the optimal cut-off value of baPWV for detection of CVD was 1,591 cm/s with a sensitivity of 72.7% and specificity of 71.6% (area under curve 0.778, 95% CI 0.64–0.91, p<0.05). Thus, a baPWV greater than 1,591 cm/s was an independent predictor of CVD in KT recipients with an odds ratio of 6.3 (Table 2).

**Table 2:** Independent predictors of cardiovascular disease.

	Univariate		Multivariate	
Variables	OR (95% CI)	p-value	OR (95% CI)	p-value
Pulse pressure	1.107 (1.048–1.169)	<0.05	1.10 (1.01–1.14)	<0.05
CVD history	9.429 (2.001–44.429)	<0.05	7.63 (1.19–48.76)	<0.05
BMI	1.222 (1.039–1.436)	<0.05	1.26 (1.03–1.55)	<0.05
baPWV>1,591cm/s	9.417 (1.928–45.986)	<0.05	6.3 (1.01–38.87)	<0.05
Age	1. 067 (0.998–1.140)	0.057		
Male sex	1.176 (0.328–4.129)	0.804		
LVMI	1.017 (1.000–1.035)	0.057		

OR, odds ratio; CI, confidence interval; CVD, cardiovascular disease; BMI, body mass index; baPWV, brachial-ankle pulse wave velocity; SBP, systolic blood pressure; LVMI, left ventricle mass index

### Association between Pre-transplant Brachial-Ankle Pulse Wave Velocity and Coronary Calcium Score

**Fig 4:**
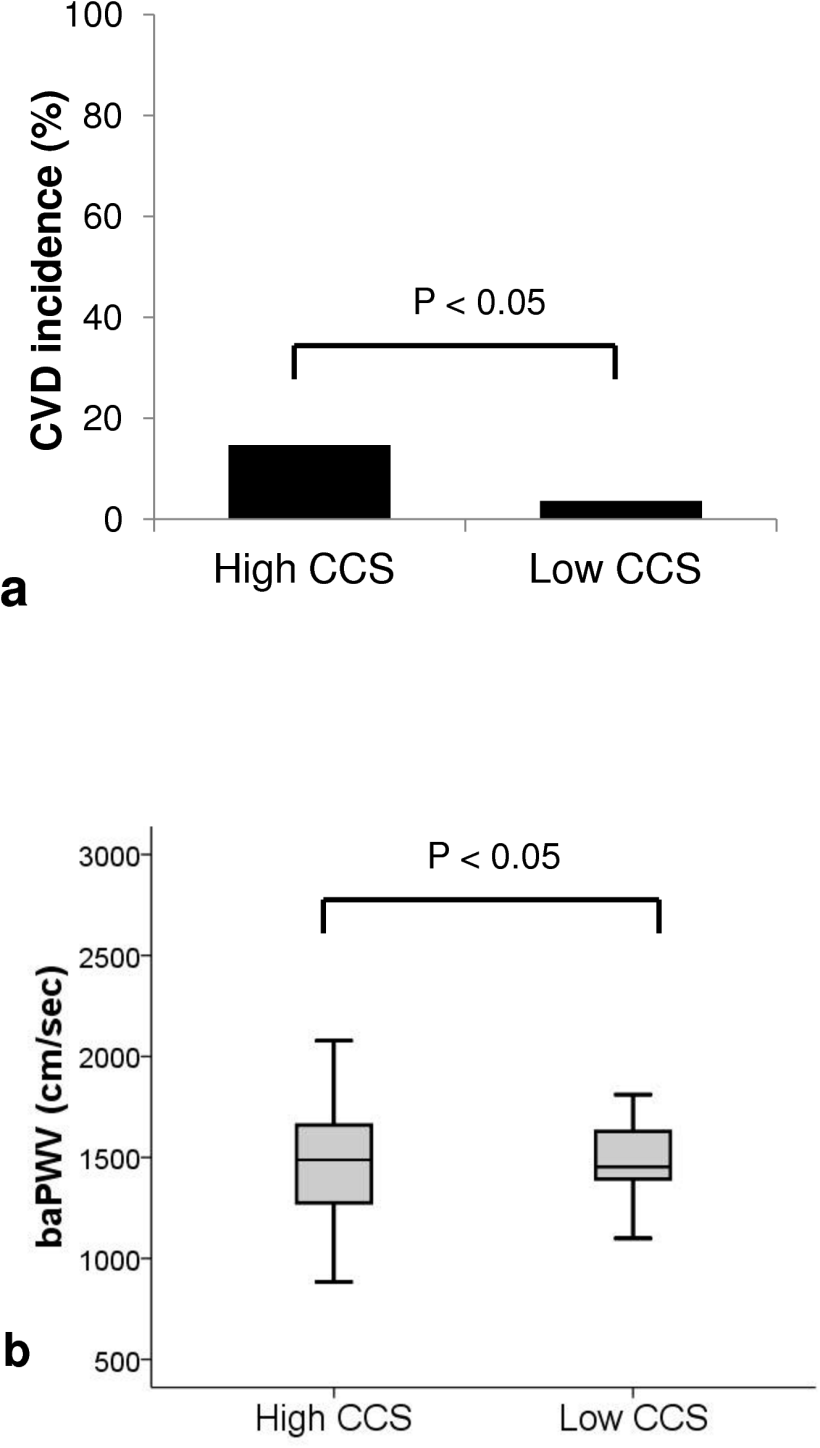
Comparison of cardiovascular disease incidence and brachial-ankle pulse wave velocity between high and low coronary calcium score. (A) CVD incidence rate. (B) baPWV. CVD = cardiovascular disease; baPWV = brachial-ankle pulse wave velocity; CCS = coronary calcium score.

As shown Fig 4A, the occurrence rate of CVD was significantly higher in those patients with a ‘high coronary calcium score’ compared to those with a ‘low coronary calcium score’ (14.7% vs. 3.6%, p<0.05). Fig 4B shows the comparison of baPWV between high and low coronary calcium score patients. The baPWV in patients with high coronary calcium score was significantly higher compared to that of patients with a low coronary calcium score (1627 ± 393 vs. 1479 ± 265 cm/s, p<0.05).

### Association between Pre-transplant Brachial-Ankle Pulse Wave Velocity and Pulse Pressure

**Fig 5:**
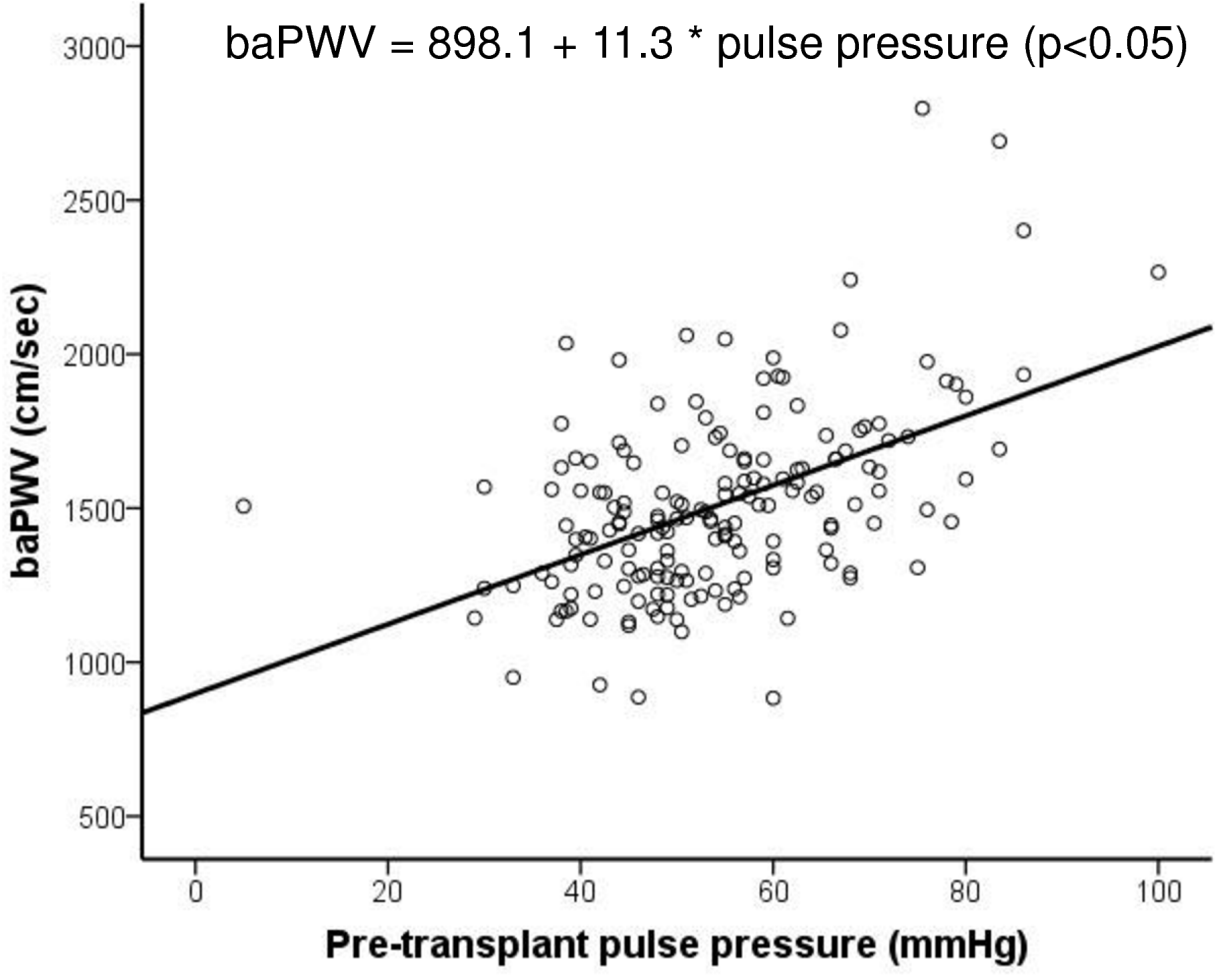
The relationship of brachial-ankle pulse wave velocity and pulse pressure. Note that baPWV increased followed as the increase of pulse pressure, and the relationship was statistically significant. baPWV = brachial-ankle pulse wave velocity.

Fig 5 shows the significant relationship of baPWV and pulse pressure. As pulse pressure increased, baPWV increased according to statistical calculation formula as shown Fig 5. (r = 0.5, r^2^ = 0.249, p<0.001).

### The Effect of Kidney Transplant on Changes in Brachial-Ankle Pulse Wave Velocity

**Fig 6:**
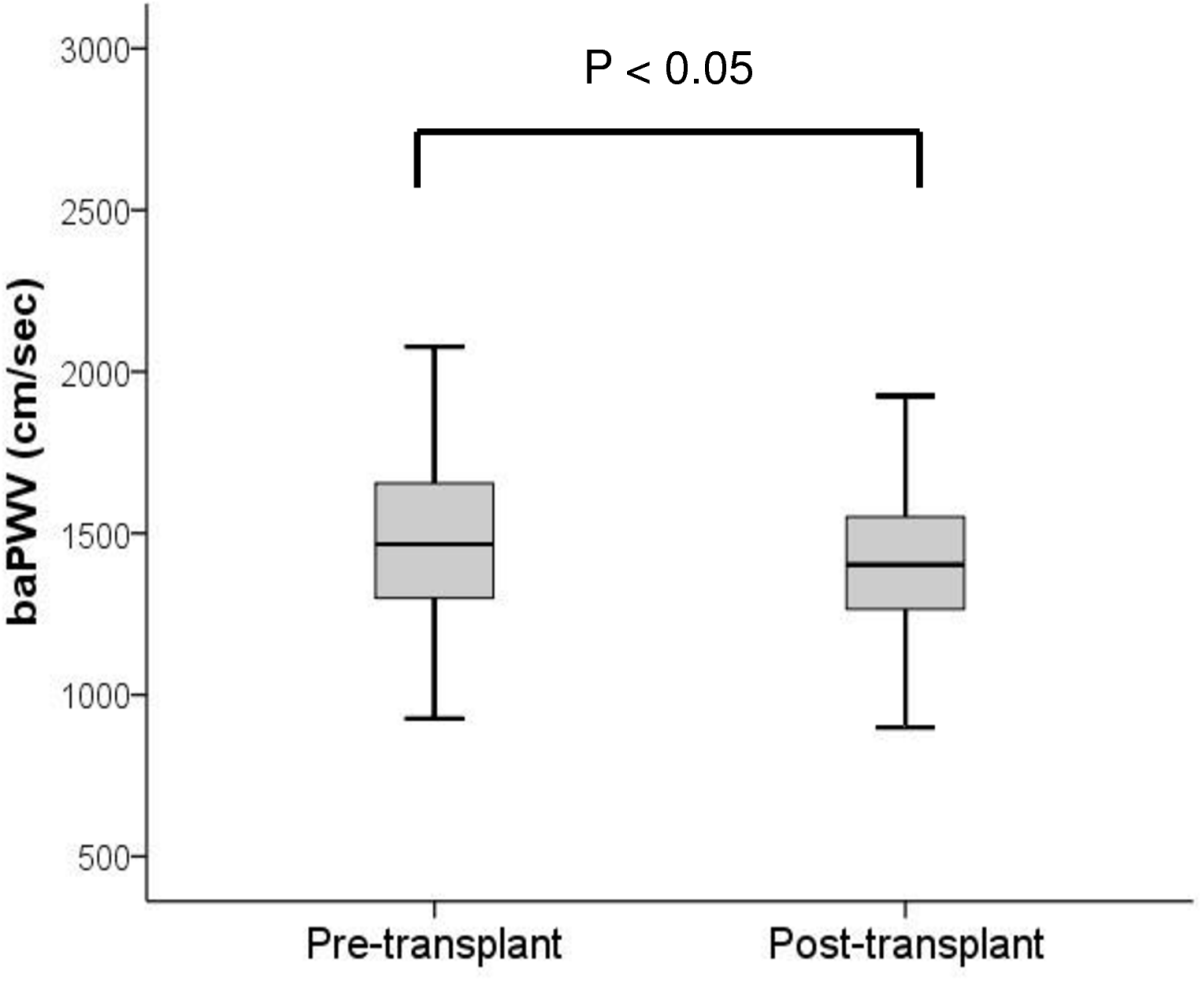
The effects of kidney transplantation on brachial-ankle pulse wave velocity in end stage renal disease patients. Note significant decrease of baPWV after KT compared to pre-transplant. baPWV = brachial-ankle pulse wave velocity; ESRD = end stage renal disease; KT = kidney transplantation.

**Fig 7:**
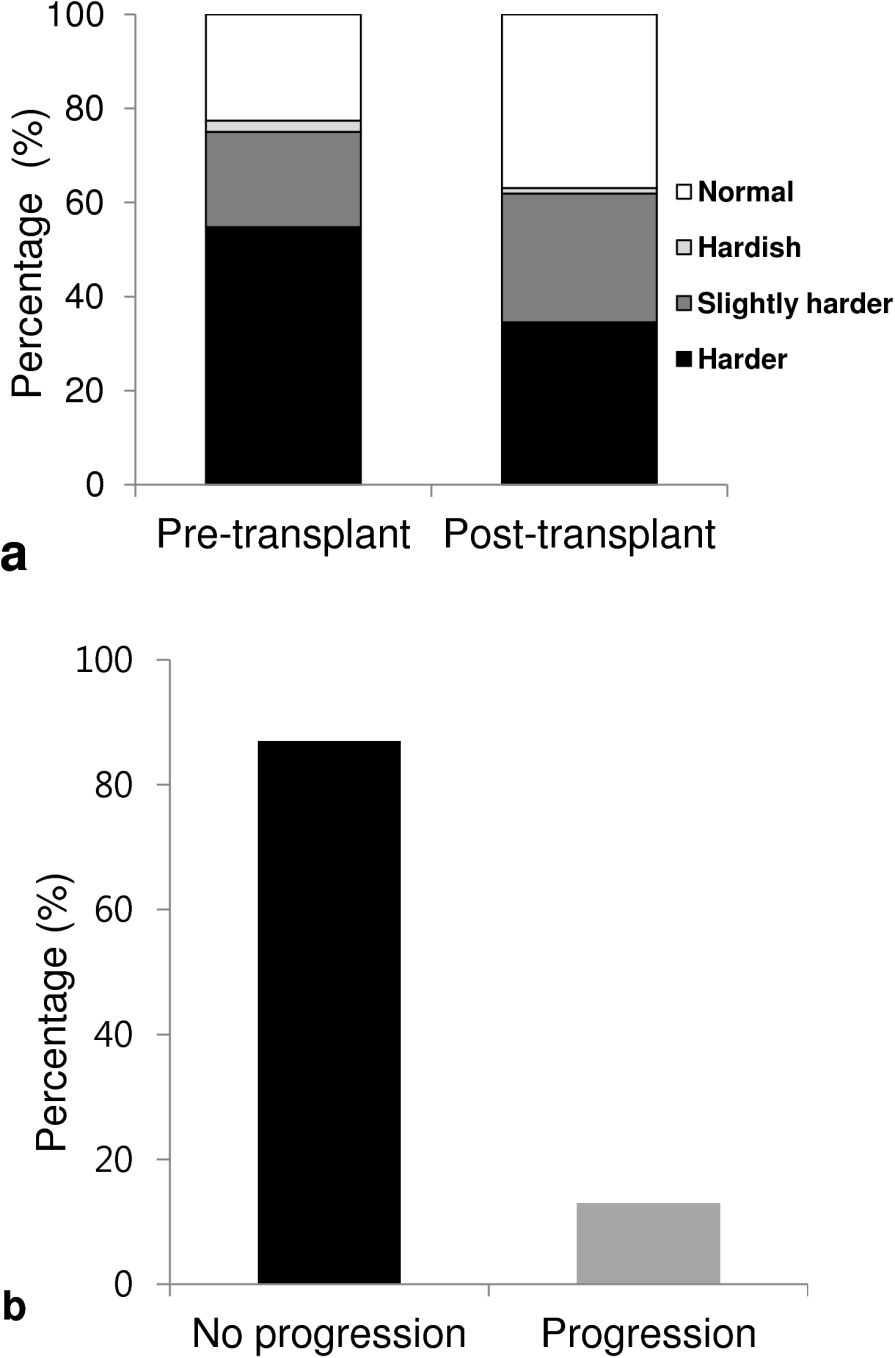
The effects of kidney transplantation on progression of arterial stiffness. (A) The changing pattern of arterial stiffness after KT. (B) The progression of arterial stiffness after KT. Note that over eighty percent of ESRD patients showed no progression of arterial stiffness after KT. KT = kidney transplantation; ESRD = end stage renal disease.

KT significantly decreased baPWV compared with the rate before KT (1418 ± 235 vs. 1517 ± 293 cm/s, p<0.05, Fig 6). After KT, the rate of the ‘normal group’ increased (22.6% vs. 36.9%), the ‘hardish group’ decreased (2.4% vs. 1.2%), the ‘slightly harder group’ increased (20.2% vs. 27.4%), and the ‘harder group’ decreased greatly (54.8% vs. 34.5%). When we evaluated this change from the point of view of progression, 86.9% patients showed no progression (with an improvement observed in 34.5%, and no change in 52.4%) and only 11 of 84 patients (13.1%) showed progression after KT (Fig 7A and 7B).

Table 3 shows the comparison between the progression and the no progression groups. Higher pre-transplant baPWV, lower body mass index (BMI), and a smaller increase in calcium levels observed in the no progression group (p<0.05).

**Table 3:** Comparison of clinical characteristics of patients with and without progression of arterial stiffness.

	No Progression (n = 73)	Progression (n = 11)	p-value
Age (year)	45.7 ± 11.6	42.4 ± 9.8	0.372
BMI (%)	22.6 ± 3.4	26.6 ± 3.9	<0.05
Pre-transplant baPWV (cm/sec)	1548 ± 986	1305 ± 363	<0.05
Post-transplant baPWV (cm/sec)	1410 ± 669	1459 ± 455	0.526
Pre-transplant Ca (mg/dL)	8.6 ± 0.6	8.2 ± 0.7	0.225
Post-transplant Ca (mg/dL)	9.3 ± 0.6	9.8 ± 0.6	<0.05
Pre-transplant Ph (mg/dL)	5.1 ± 1.6	5.5 ± 0.9	0.468
Post-transplant Ph (mg/dL)	3.5 ± 0.5	3.8 ± 0.4	0.078
Ca delta	0.7 ± 1.1	1.6 ± 0.8	<0.05
Pre-transplant Pulse pressure (mmHg)	57.3 ± 14.1	55.6 ± 13.6	0.709
Pre-transplant EF (%)	60.3 ± 5.2	62.7 ± 3.7	0.149
Pre-transplant LVMI (g/m^2^)	145.5 ± 40.0	120.4 ± 15.3	0.178

BMI, body mass index; baPWV, brachial-ankle pulse wave velocity; KT, kidney transplantation; Ca, calcium; Ph, phosphate; EF, ejection fraction; LVMI, left ventricle mass index

### Risk Factors for Changing Brachial-Ankle Pulse Wave Velocity after Kidney Transplantation

Table 4 shows the logistic regression analysis of risk factors that affected the baPWV after KT. The degree of increase in serum Ca level and BMI associated with changes in baPWV (p<0.05, Table 4).

**Table 4:** Risk factor analysis for prediction of brachial-ankle pulse wave velocity progression.

	Univariate		Multivariate	
Variables	OR (95% CI)	p-value	OR (95% CI)	p-value
BMI	1.321 (1.099–1.589)	<0.05	1.348 (1.049–1.732)	<0.05
Ca delta	2.752 (1.259–6.016)	<0.05	4.255 (1.492–12.132)	<0.05
SBP	1.043 (1.007–1.079)	<0.05	1.068 (0.970–1.176)	0.066
DBP	1.065 (1.009–1.124)	<0.05	1.018 (0.882–1.175)	0.602
Sex	0.526 (0.142–1.954)	0.337		
Age	0.975 (0.924–1.030)	0.370		
CVD history	1.27 (1.00–1.55)	0.198		

OR, odds ratio; CI, confidence interval; BMI, body mass index; Ca, calcium; SBP, systolic blood pressure; DBP, diastolic blood pressure; CVD, cardiovascular disease

## Discussion

The results of our study clearly demonstrate that pre-transplant baPWV was closely associated with CVD incidence in KT recipients. After KT, baPWV was significantly reduced, and the severity of arterial stiffness was improved. This finding suggested that measurement of baPWV could represent a useful screening test to predict CVD after KT, and KT is effective in preventing the progression of arterial stiffness in ESRD patients.

It is well known that arterial stiffness increases in ESRD patients, and arterial stiffness is closely associated with CVD incidence in ESRD patients. [7, 14–16] The results of our study showed that 93.6% of ESRD patients showed higher baPWV than healthy controls and that high pre-transplant baPWV was a strong predictive factor of CVD after KT (OR 1.003, CI: 1.001–1.005, p<0.05). In KT patients with CVD, pre-transplant baPWV was higher than that of patients without CVD (1800 ± 440 vs. 1491 ± 265 cm/s, p<0.05, Fig 2). This result confirms previous studies that ESRD patients with higher baPWV rates are prone to develop CVD after KT. [8, 17]

The baPWV value is influenced by several factors [8, 18, 19]. Therefore, we calculated the cut-off value for baPWV as a predictor of CVD. The ROC curve analysis revealed that the optimal cut-off value of pre-transplant baPWV for detection of CVD was 1,591 cm/s. As shown Table 2, A baPWV value greater than 1,591 cm/s had an odds ratio of 6.3, and was a strong independent predictor of CVD even after adjusting for confounders including age, sex, CVD history, and BMI, LVMI, pulse pressure (Table 2). Therefore, the cut-off value for baPWV may be beneficial to clinicians in modifying immunosuppression therapy or in attenuating risk factors of CVD after KT.

There are many non-invasive methods for detecting cardiovascular status before KT, and each has its own merits and faults. In our hospital, we used 24-hour ambulatory blood pressure, pulse pressure, echocardiogram, coronary calcium score, and baPWV for assessing accurate cardiovascular conditions. In this study, we evaluated the effectiveness of baPWV by comparing the coronary calcium score and pulse pressure [12, 20–24]. As a result, the baPWV value was significantly higher in the high coronary calcium score group with scores over 100, and has significant relationship with pulse pressure. The baPWV was not only closely associated with coronary calcium score but also pulse pressure which are well-known predictor of CVD. This finding suggested that measurement of baPWV as well as that of coronary calcium score and pulse pressure are suitable diagnostic tools for predicting CVD. [20, 24, 25]

It is recognized that an accelerated progression of arterial stiffness occurs in dialysis patients compared with the general population. [5, 26] In our study, we evaluated whether KT could prevent progression of arterial stiffness, and found that post-transplant baPWV was significantly lower compared to pre-transplant rates (1,418 ± 235 vs. 1,517 ± 293 cm/s, p<0.05). We found that 86.9% patients showed no progression (Fig 7B), which suggested that KT is effective in preventing the progression of arterial stiffness in over 80% of ESRD patients. However, more than half of KT patients were still within the abnormal range even after KT, and 13.1% patients progressed after KT. This finding implies that arterial stiffness is still worse in KT than in healthy controls, and the risk of CVD events was still higher than healthy controls even after KT. Therefore, we need to pay special attention to KT recipients who show progression of arterial stiffness.

The effects of KT on arterial stiffness showed controversial results, and this appeared to be related to several factors such as the study population, evaluation time points, and the interpretation of pulse wave velocity results. Compared to previous studies, our study presents some peculiarities. First, the study population was relatively young (44.5 ± 11.6 years), [7, 8] and the mean dialysis period (32.6 months) was shorter than in other studies, while the proportion of non-dialysis patients (24%) was higher than in other studies. [8, 27] These factors may explain the lower incidence of CVD observed in our study than in previous reports. [6, 7] Second, we measured baPWV one year after KT. Most previous reports evaluated baPWV at a perioperative time within 6 months after KT. [8–10] The reason for choosing one year after KT was to exclude the effect of the immunosuppression, and one year after KT is an important time point to predict long-term graft and patient survival. [28, 29] Third, we interpreted pulse wave velocity results as progression of arterial stiffness. Previous reports interpreted baPWV after KT as change of baPWV. [6–8] Thus, it was difficult to apply these results directly in the clinic. Therefore, we classified patients into four groups according to the severity of arterial stiffness, and interpreted post-transplant baPWV change as progression versus non-progression.

We further evaluated the factors influencing changes in baPWV following KT, and two factors (the increase in calcium levels and BMI) resulted to be involved in baPWV progression (Table 4). It is well known that elevated calcium is an early marker of arteriosclerosis, [30] and high intake of supplemental calcium increases baPWV and cardiovascular disease [19] by increasing vascular calcification with synergistic effects by serum Ph. [31] The rise in the BMI was also strongly related to increased pulse wave velocity. [32] It is well known that adipocytes trigger insulin resistance, increase small dense LDLs, and stimulate inflammation, which are important reactions in atherosclerosis. [33, 34] These two factors are correctable with better patient education, and clinicians recommended to control patient’s serum Ca levels and body weight to achieve better outcomes, and controlling these two may be helpful to prevent CVD by decreasing baPWV progression.

This study has some limitations. First, we did not screen post-transplant baPWV in all patients as a routine procedure. Second, the total number of CVD and baPWV progression observed was too low (n = 10, n = 11). Third, follow up duration (33.9 months) was too short to evaluate long-term CVD. Fourth, we did not have a data of arterial reactivity or endothelial dysfunction (flow-mediated dilatation (FMD)). In fact, there are many reports about endothelial dysfunction in chronic kidney disease (CKD) patients and KT recipients, and they represented that CKD patients have decreased FMD, and it worsening with increasing CKD, and improved after KT [35].

Despite these limitations, the results of our study suggested that baPWV can be used as one of the screening tests for predicting CVD in KT recipients.

In conclusion, our data indicates that higher pre-transplant baPWV is a predictor of CVD in KT recipients, and KT deters the progression of arterial stiffness. Therefore, we recommend baPWV as a screening tool before KT. Follow-up baPWV is necessary to understand the progression of arterial stiffness after KT.
